# Embryology training for Reproductive Endocrine fellows in the clinical human embryology laboratory

**DOI:** 10.1007/s10815-014-0189-0

**Published:** 2014-02-21

**Authors:** Richard T. Scott, Kathleen H. Hong, Marie D. Werner, Eric J. Forman, Andrew Ruiz, Michael C. Cheng, Tian Zhao, Kathleen M. Upham

**Affiliations:** 1 Reproductive Medicine Associates of New Jersey, 140 Allen Road, Basking Ridge, NJ 07920 USA; 2 Division of Reproductive Endocrinology, Department of Obstetrics Gynecology and Reproductive Science, Robert Wood Johnson Medical School, Rutgers University, New Brunswick, NJ USA

**Keywords:** Reproductive Endocrine fellowship training, Embryology, Embryology laboratory, IVF

## Abstract

**Objective:**

To determine if comprehensive embryology training for clinical Reproductive Endocrinology fellows could be completed to a level of proficiency equivalent to that of experienced embryologists.

**Method:**

Clinical fellows were integrated into the clinical embryology team and were trained to perform all the various procedures utilized in clinical embryology. The fellows were trained to the same standards as the clinical embryology staff and underwent the same certification and sign off procedures. To determine if inclusion of clinical fellows on the embryology team impacted outcomes, outcomes for individual oocytes/embryos and the clinical cases where the fellows perform embryology procedures were compared to the outcomes of those oocytes/embryos and cases performed by the full time embryology staff.

**Results:**

Clinical procedures performed by the fellows included isolation and processing of oocytes following retrieval, loading catheters for embryo transfer, and vitrification (*N* = 823 cases). Micromanipulation procedures compared included ICSI and assisted hatching (*N* = 650 cases). For each procedure, the outcomes in those cases performed by the RE fellows were equivalent to those done by the fully trained clinical embryology staff.

**Conclusions:**

When fellows are trained to perform embryology procedures as an integral part of their fellowship curricula, laboratory efficiencies and clinical outcomes are fully maintained. This experience provides valuable insight into the ART process critical to this subspecialty. It also empowers fellows to fully participate in research relating to the viability of gamete and embryos and optimization of the clinical ART laboratory.

## Introduction

Reproductive Endocrine (RE) training is near unique amongst medical specialties in that the expected breadth of knowledge extends far beyond clinical care. Extensive expertise with developmental biology from the gamete level to full maturity and then to senescence is expected. Significant knowledge in the areas of pediatric and medical endocrinology, while outside of clinical practice for virtually all Reproductive Endocrinologists, is also required [1]. Perhaps most distinctive is the focus on basic and laboratory science. Fellowship graduates are expected to have comprehensive knowledge of a variety of techniques stemming from basic hormonal assays to newer molecular techniques. This is in spite of the fact that very few of these individuals will ever return to the laboratory to actually perform these analyses themselves after completing fellowship training.

The reason such a broad scientific foundation is required is clear and unassailable. Reproductive Endocrinology requires a fundamental understanding of each of these processes to optimize clinical practice, critically review new medical and scientific literature, and to empower graduates to become scientists capable of meaningful investigation. Hands on experience provides valuable insight and understanding about performing and interpreting research even if that particular laboratory assay or process is not routinely used in their future clinical care or research efforts.

Over the last three decades, the development of the assisted reproductive technologies (ART's) has led to their becoming the treatment of choice for a substantial portion of infertile couples. Recent studies have clearly demonstrated that they are the most successful and cost effective approaches for many patients requiring clinical care [2, 3]. A significant component of the ART's are the embryology and andrology laboratories.

In sharp contrast to every other area of Reproductive Endocrinology, fellowships typically provide little actual embryology training. Reproductive Endocrinology fellowship programs are required to provide exposure, but this standard may be met in widely differing ways. Anecdotally, many programs meet this requirement by having the fellow observe at a distance within the laboratory while not participating in the actual processes. Others may not allow the fellows in the laboratory at all. A few programs provide an opportunity to interact meaningfully with the laboratory or provide fellows an optional "training track" which would allow them to participate in these laboratories.

The reasons why training in embryology and andrology laboratories is limited while training extensively in other laboratory sciences involved in clinical Reproductive Endocrinology are varied and are not specifically quantitatable. One reason might be that having clinical fellows perform embryology procedures in the actual clinical laboratory would compromise patient care. In reviewing this premise, there are no data to support such a supposition. In turn, there are no data demonstrating that it is safe.

This study seeks to determine if Reproductive Endocrine fellows may receive comprehensive embryology and andrology laboratory training in the clinical laboratories, up to and including the most sophisticated micromanipulation procedures, to a level of proficiency equivalent to that of experienced embryologists.

## Materials and methods

Reproductive Endocrinology fellows in the Robert Wood Johnson fellowship program are required to spend 6 months in the clinical embryology and andrology laboratories. This is a relatively new fellowship program. Three fellows have progressed through their training and all three have completed their laboratory rotations to date. During that time, they undergo technical training and testing which is identical to that received by the clinical embryology and andrology staff as they complete their training and certification. The fellows are tested to the same standards as clinical embryologists prior to being allowed to independently perform clinical procedures.

The fellows initially work with discarded biologic material. They spend an enormous number of hours moving oocytes which have failed to fertilize, hatching embryos which have arrested in development, and vitrifying and warming arrested embryos which are not suitable for either transfer or cryopreservation. The amount of time spent learning and mastering the technical aspects of each task was tracked for each task and for each individual fellow. Once technical proficiency has been attained, the fellow is signed off by the supervisor responsible for that area of the laboratory. They are then placed into the clinical embryology task rotation and perform actual clinical cases. An estimate from the senior embryology staff is that they spent approximately 600 h performing training on the various techniques before doing actual clinical cases.

Training was systematic and followed a plan sequence but not a specific time table. Competence at one task was required before training was begun on another. Just as with any embryologist, the fellows commonly performed clinical procedures for a task for which they had already been signed off while they trained at a different time of day on another. For example, they might be the embryologist responsible for retrievals in the morning while training for hyaluronidase/stripping prior to ICSI later in the day. The general training sequence was QA/QC, chart review and preparation, daily schedule and task list, andrology, retrievals, insemination, fertilization checks, vitrification including warming, oocyte stripping prior to ICSI, assisted hatching, ICSI, embryo biopsy, and embryo grading. During training, a qualified embryologist supervised the fellows at all times. After being signed off on a given task, the fellow had the same autonomy as any of the embryologists certified for that particular task.

It should be noted that prior to training in embryology that the fellows rotate through the andrology and endocrinology laboratory and gain proficiency in those laboratories and are signed off to perform those procedures to rotating through the embryology laboratory. The fellows do perform andrology procedures on IVF specimens during their embryology laboratory training.

### Gametes and embryos

The subjects in the study are composed of the oocytes and embryos which are undergoing culture and procedures within the clinical IVF laboratory. The exposure relates to the embryologist doing the procedure. There are two possible exposures since procedures were performed either by the clinical Reproductive Endocrinology fellows during their rotation or by the clinical embryology staff.

### Experimental design

The fellows rotate through every workstation within the laboratory. This includes daily QA/QC, chart review and preparation, creation of laboratory treatment plans, and dish preparation. These tasks are essential to good laboratory function, but the results are difficult to quantitate and were not included in the study. This study focuses on endpoints which are more directly evaluable. Specifically, it focuses on the level of proficiency attained by the fellows for performing hands on embryology procedures involving actual manipulation of the spermatozoa, oocytes, and embryos. These specific tasks were divided into three groups.

The first group of procedures relate to identifying, processing, and moving specimens within the laboratory. The initial comparison focused on performing identification and handling of the oocyte cumulus complexes at the time of retrieval. The endpoints measured related to the time spent performing these procedure. Time intervals for isolation of the oocyte cumulus complexes, cutting and removing excess cumulus, and the total time for the procedure which includes the first two steps as well as the time spent to transfer the oocytes into their initial culture dishes and place them in an incubator. Effectiveness in loading the catheter at the time of embryo transfer was measured by assessing pregnancy rates which ensued from those transfers.

The second group of procedures related to embryo vitrification. Two important endpoints were assessed. The first was the efficiency with which the initial vitrification was accomplished. This was evaluated by comparing the proportion of embryos which survived warming. This step assessed only which group did the actual vitrification. It did not matter if the warming phase was done by a fellow or a clinical embryologist. The second endpoint was the pregnancy outcome following transfer of those warmed embryos.

The third group of procedures involved micromanipulation. Two procedures were evaluated- ICSI and assisted hatching. The outcomes following ICSI were evaluated by comparing the fertilization rate, the eventual proportion of injected oocytes which formed blastocysts of sufficient quality to be transferred or be vitrified for future use, and the pregnancy rate which ensued following fresh transfer of those blastocysts. Skill with assisted hatching was similarly evaluated by assessing the proportion which progressed to become high quality blastocysts and the pregnancy rates for those which were transferred.

### Data analysis

All data within the clinical laboratory are tracked real time in an electronic medical record. Data consistently recorded in the electronic record include the individual performing the procedure and the results of that procedure. These data, as well as the clinical outcomes for the overall cycle were extracted from the electronic medical record as appropriate.

The primary endpoints for each of the procedures were compared using Student’s t-tests for continuous variables or contingency table analyses for categorical variables as appropriate. An alpha error of 0.05 was considered significant. No power analysis was done. Study size was determined by capturing 100 % of the procedures performed by the RE fellows during their training rotation. Age matched controls were selected for each case or function based on the subsequent five cases performed by the clinical embryology team providing a 1:5 ratio for this case:control study. The 1:5 ratio was selected arbitrarily as it provided a large number of control procedures to enhance the precision of the statistical result, but was limited to procedures which were typically performed within 1 day of those done by the clinical RE fellows. This limit was felt to be prudent to minimize any impact of non-specific laboratory drift.

There was no evaluation of longitudinal changes in the performance of the fellows with the various embryology tasks. This was not practical as the number of procedures being done was still relatively limited and there was insufficient power to perform this type of analysis. This retrospective study was IRB approved.

## Results

Three fellows have completed their rotations to date. All fellows successfully completed their training rotation in every area within the laboratory and were able to meet the established performance standards required to be signed-off to function autonomously. All data regarding the procedures performed by the fellows and the subsequent outcomes were recorded and are reported. No procedures or results have been removed. The list of the procedures which were performed, the numbers of each performed by the RE fellows, and the number of controls (always selected on the basis of a 1:5 ratio) are listed in Table 1.

**Table 1:** Types and number of procedures performed by clinical RE fellows and their age matched controls done by the clinical embryology team

		Procedures performed by:	
		Reproductive Endocrine fellows	Clinical embryologists
Gamete embryo handling			
	Oocyte-cumulus complexes recovered and processed at time of retrieval (cases)	200	1,000
	Fertilization check the day following insemination (cases)	173	865
	Catheters loaded for embryo transfer (cases)	123	615
Vitrification			
	Embryos vitrified and subsequently warmed (N)	141	705
	Number of Embryo Transfers (cases)	186	930
Micromanipulation			
	ICSI		
	Cases	250	1,250
	Oocytes Injected	2,248	11,240
	Assisted Hatching		
	Cases	212	1,060
	Embryos	1,077	5,385

**Fig. 1:**
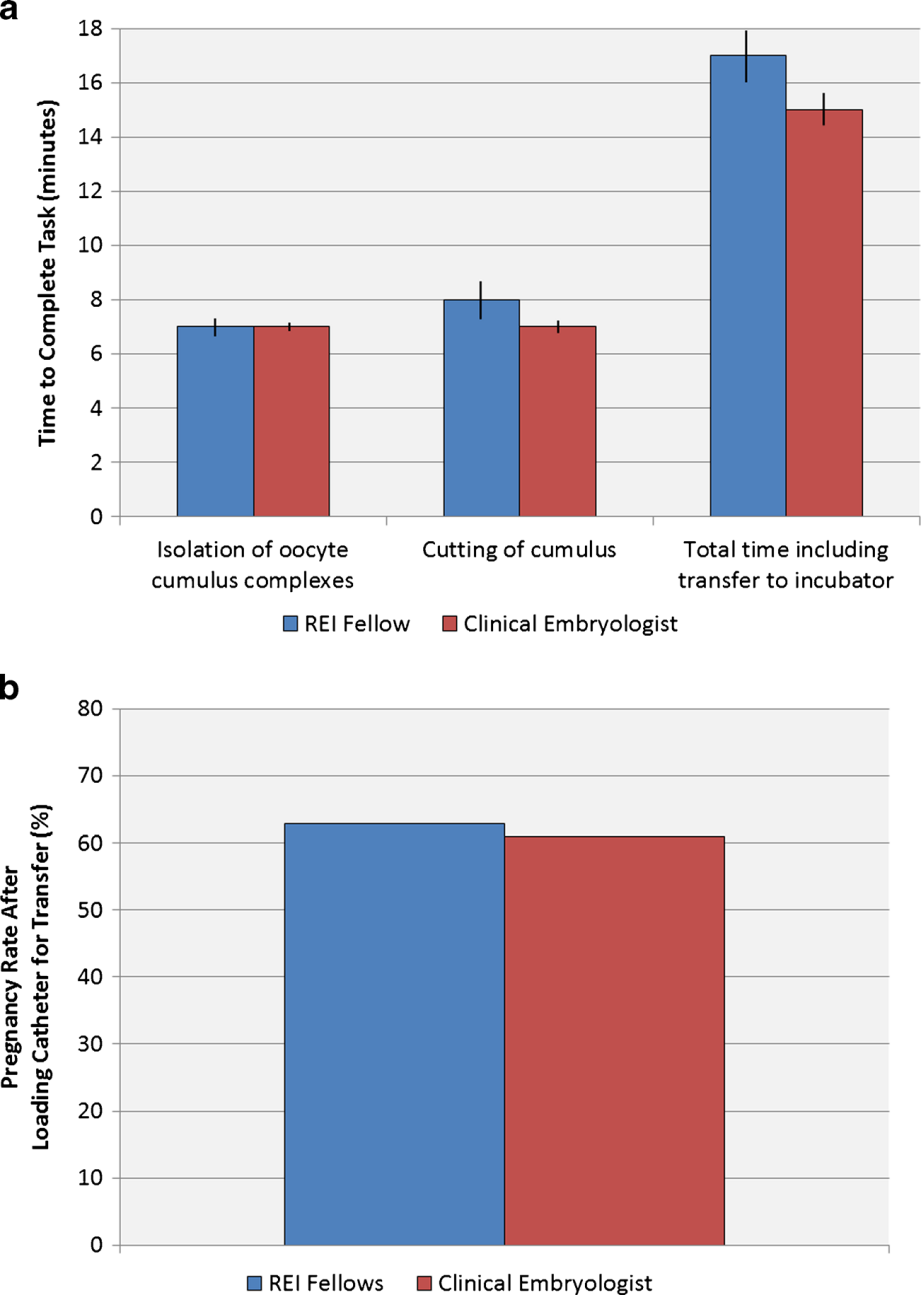
The ability of Reproductive Endocrine fellows to isolate and manipulate gametes and embryos was assessed. Isolation and processing of oocytes at the time of oocyte retrievals was measured by looking at the time interval to identify the oocyte cumulus complexes, remove the excess cumulus, and then the total time for the entire procedure including washing the recovered oocytes, transferring them to a culture dish and then placing them in an incubator (**a**). There were no differences between the RE fellows and the clinical embryology team. Pregnancy rates after loading the catheter at the time of embryo transfer were also equivalent for the RE fellows and clinical embryologists (**b**)

The first group of procedures to be evaluated related to isolating and processing oocyte cumulus complexes at the time of retrieval or loading embryos for transfer to the patient at completion of the in vitro process. There were no differences in the time spent to recover and process the oocytes immediately following retrieval. Specifically, the time spent looking through the follicular aspirates and isolating the oocyte cumulus complexes (*P* = 0.92), the time spent cutting excess cumulus away from the oocyte (*P* = 0.72), and the total time from onset of the retrieval until recovery was complete with the oocytes put away in an incubator were all equivalent (*P* = 0.52) (Fig. 1a). Similarly, pregnancy rates were not impacted by whether the catheter was loaded by the fellow or by a member of the clinical embryology staff (*P* = 0.77) (Fig. 1b).

**Fig. 2:**
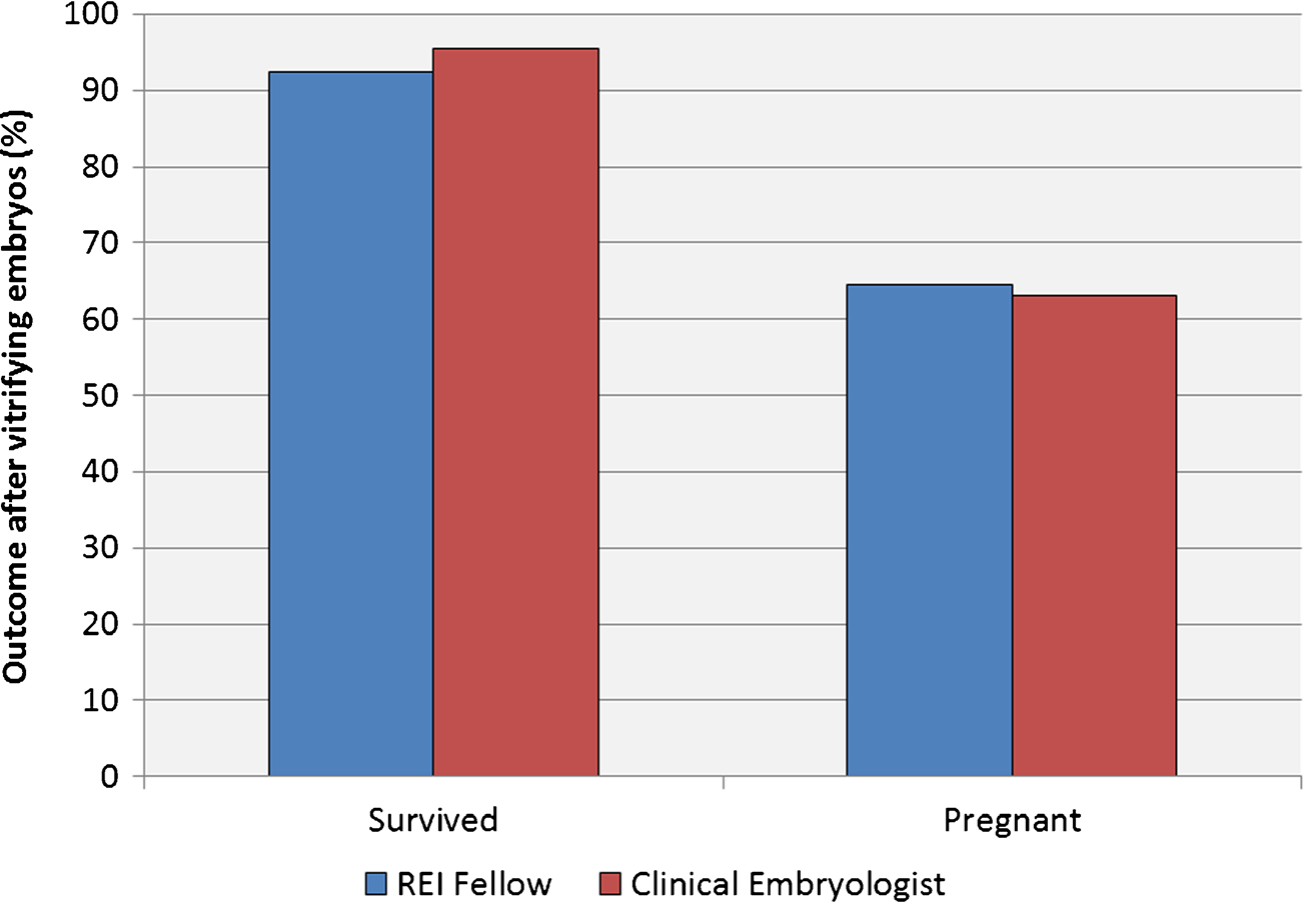
When evaluating embryos which were vitrified by either the RE fellows or the clinical embryology team, there were no differences in the survival rates after warming or in the subsequent pregnancy rates following transfer

The second group of procedures dealt with vitrification. There were no differences in survival for embryos vitrified by the fellows versus those vitrified by the clinical embryologists (*P* = 0.22). Those embryos were then transferred. Similarly, the ensuing pregnancy rates were equivalent for those embryos vitrified by the fellows or the clinical embryologists (*P* = 0.85) (Fig. 2). The number of embryos in those transfers were equivalent (1.7 versus 1.8) (*P* = 0.92) and the implantation rates were also equivalent (53 % vs 49 %) (*P* = 0.44) for those originally vitrified by the RE fellows and embryologists, respectively.

**Fig. 3:**
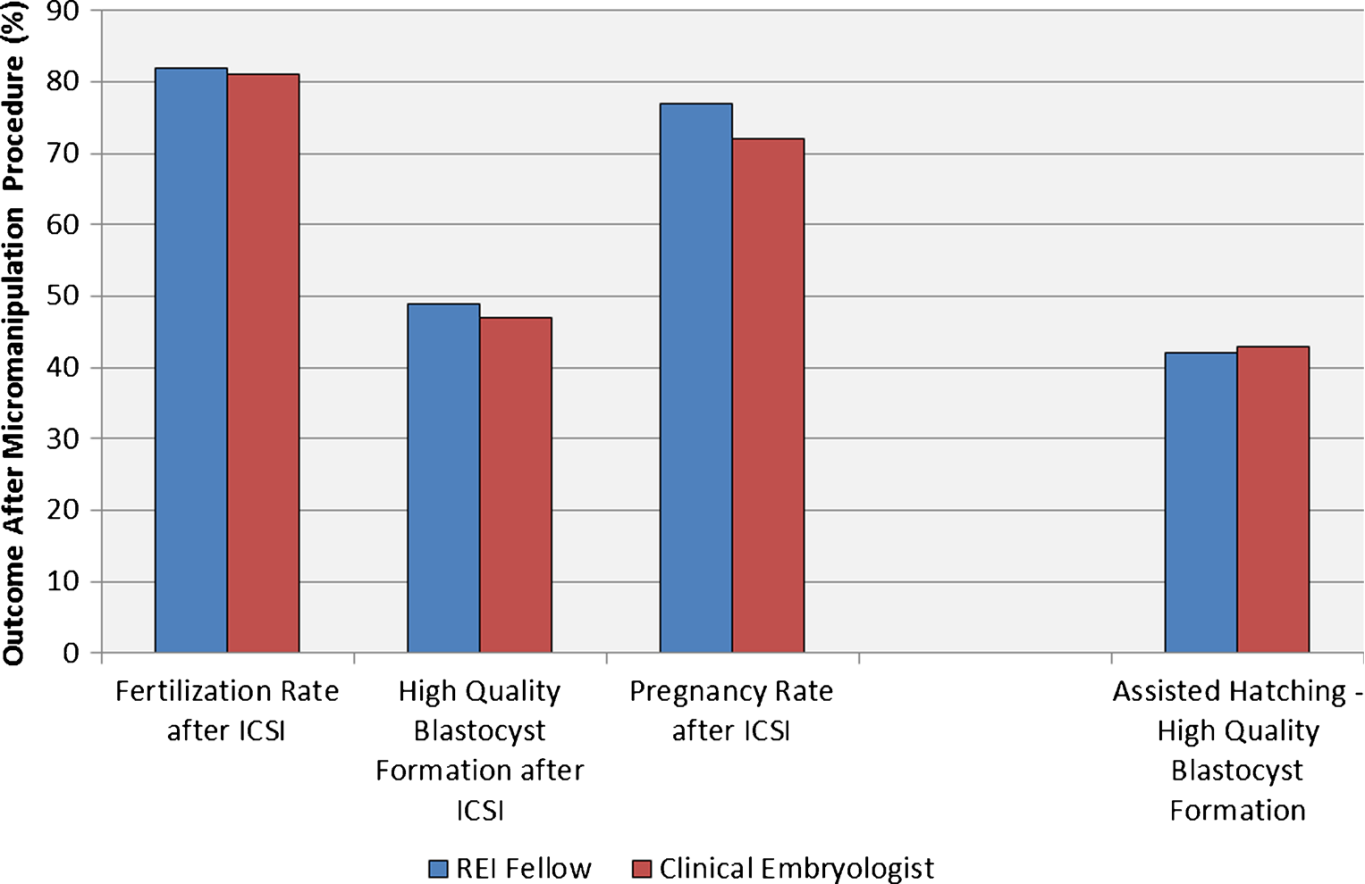
Micromanipulation: Proficiency with micromanipulation was assessed by comparing outcomes for those procedures performed by the RE fellows relative to the clinical embryology staff. For ICSI, there were no differences in fertilization rates, the proportion of embryos which formed high quality embryos, or the pregnancy rates which ensued following transfer. Similarly, outcomes following assisted hatching were equivalent for both the fellows and the embryologists

The final set of evaluations addressed the various micromanipulation procedures done in the embryology laboratory. Following ICSI, there were no differences in fertilization rates (*P* = 0.89), the proportion of the injected oocytes which became high quality blastocysts (*P* = 0.78), or pregnancy rates following transfer (*P* = 0.54) (Fig. 3a).

In this program, embryos are hatched on day 3 and placed into extended culture. Development is observed again on day 5 and if the embryos are of sufficient quality they are either transferred or considered supernumerary and vitrified for future use. The proportion of embryos which blastulated following assisted hatching was the same for the RE fellows and the clinical embryologists (*P* = 0.6) (Fig. 3b).

In summary, the outcomes for procedures performed by the RE fellows were equivalent to those done by the fully trained and credentialed clinical embryology staff for all procedures evaluated.

## Discussion

This study demonstrates that it is possible to provide clinical embryology training to Reproductive Endocrine fellows as an integral component of their fellowship. The fellows attained a high level of proficiency with the full spectrum of laboratory procedures. Most importantly, their training was completed in the clinical embryology laboratory and they participated in the care of actual clinical cases without compromising clinical results.

Incorporation of comprehensive embryology training into the fellowship program did not require a reduction in any aspect of the fellows’ clinical training. They spent the full amount of time prescribed by the American Board of Obstetrics and Gynecology on clinical rotations and saw the same number of patients, performed the same number of surgeries, and completed the same number of office related procedures as they would have had they not been rotating through the embryology laboratory. There is absolutely no compromise in any aspect of clinical training.

The embryology laboratory in many programs is a sacred and even mysterious place with extremely limited access. The thought of conducting RE fellow training in this setting is daunting. In reality, it is no different than many areas of training. Thoughtful training algorithms and close supervision are required. It is wholly analogous to teaching young physicians to do deliveries, perform surgeries, and even do procedures such as oocyte retrievals or embryo transfers. This study demonstrates that meaningful training may be completed without compromising clinical results, functionally lifting the mysterious veil which surrounds these laboratories.

No additional resources should be required when training Reproductive Endocrine fellows within the embryology and andrology laboratories. Given that there are no specific graduate programs in human embryology and andrology that provide hands-on technical training, embryology skills are typically attained in a manner which might best be characterized as an apprenticeship. As such, almost all programs have the algorithms and resources in place which allow them to train and sign-off individuals to perform the various tasks required in clinical embryology.

The fellows have put their training to use and are continuing to work in the clinical IVF laboratory performing procedures at all levels which relate to a variety of their research projects. With this ongoing involvement, these fellows will have a sufficient number of cases to meet the volume of procedures required to become a High Complexity Laboratory Director (HCLD) as designated by the American Board of Bioanalysts. Also with ongoing involvement, they will soon have sufficient tenure in the laboratory to make them eligible to sit for the certifying examination to become HCLD’s. To be clear, there is no requirement that Reproductive Endocrinologists become qualified to be embryology or andrology laboratory directors. Rather, it speaks to the fact that the training is sufficient to meet the most rigorous regulatory requirements applied to individuals who work in leadership positions within laboratories in this field.

The putative benefits of a strong background in embryology and andrology are many. A greater understanding of the inner workings of the laboratory should facilitate better communication with the clinical embryology team. It would also allow these physicians to take a more active role in QA and QC in the laboratory throughout their careers. This is particularly important for many who will become program directors as they should be involved in all aspects of their ART program.

It may also facilitate research. Reproductive Endocrine training allocates a significant amount of time within the fellowship for research. In general, this protected time allows fellows with laboratory based projects to go into the laboratory and gain actual proficiency with both the technical and theoretical aspects of sample preparation as well as performance of the assays used to evaluate them. To attain an equivalent level of involvement for projects involving gametes or embryos, fellows must participate in the care of the embryos (intervention studies) or at least collection of biologic specimens (studies of discarded material).

The significant technical proficiency in handling and processing spermatozoa, oocytes, and embryos as well as with embryo vitrification and the performance of sophisticated micromanipulation procedures empower fellows to fully participate in any projects involving gametes or embryos. With rapid development of the “Omics” technologies, a strengthened focus has emerged on evaluating gametes, embryos, cumulus, and spent culture media [4–7]. The attained technical skills have already facilitated translational research. The fellows are providing *clinical and embryology* care in five randomized clinical trials at the current time.

Not having an individual fellow attain these skills does not preclude them from meaningful participation in research related to gametes or embryos. Rather, development of the core skills sets described in this study might empower them to participate more fully in those types of projects. Hopefully having this group of young physicians develop expertise across the full breadth of reproductive biology, laboratory science, and clinical Reproductive Endocrinology should be empowering if their research focus deals with any aspect of clinical or basic science gamete or embryo biology.

Should there be a role for embryology training in all fellowships? It is not a technical requirement at this time and there are a great number of outstanding Reproductive Endocrinologists who did not have this type of training during their fellowship. In spite of that, it is the opinion of the authors that participating in the laboratory will make it easier for Reproductive Endocrinologists to become outstanding investigators in these areas.

At the current time, the data from this study demonstrate that it is possible to safely provide embryology and andrology training without adversely impacting clinical outcomes for the patients receiving care within the division. This should empower training in clinical embryology – especially for those fellows pursuing ART related research projects or those who will choose to focus on ART during their career.

## References

[1] American Board of Obstetrics and Gynecology. Guide to learning for reproductive endocrinology. Available at http://www.abog.org/publications/REI-GUIDE-2004.pdf Accessed 27 May 2013

[2] Reindollar RH, Regan MM, Neumann PJ, Levine BS, Thornton KL, Alper MM (2010). A randomized clinical trial to evaluate optimal treatment for unexplained infertility: the fast track and standard treatment (FASTT) trial. Fertil Steril.

[3] Reindollar RH, Thornton KL, Ryley D, Alper MM, Fung JL, Goldman MB (2011). A randomized clinical trial to determine optimal infertility therapy in couples when the female partner is 38–42 years: preliminary results from the forty and over infertility treatment trial (FORT-T). Fertil Steril.

[4] Forman EJ, Hong KH, Ferry KM, Tao X, Taylor D, Levy B, Treff NR, Scott RT Jr. In vitro fertilization with single euploid blastocyst transfer: a randomized controlled trial. Fertil Steril. 2013;100:100–7. 10.1016/j.fertnstert.2013.02.056 23548942

[5] Garrido-Gómez T, Ruiz-Alonso M, Blesa D, Diaz-Gimeno P, Vilella F, Simón C (2013). Profiling the gene signature of endometrial receptivity: clinical results. Fertil Steril.

[6] Scott R, Seli E, Miller K, Sakkas D, Scott K, Burns DH (2008). Noninvasive metabolomic profiling of human embryo culture media using Raman spectroscopy predicts embryonic reproductive potential: a prospective blinded pilot study. Fertil Steril.

[7] Katz-Jaffe MG, McReynolds S, Gardner DK, Schoolcraft WB (2009). The role of proteomics in defining the human embryonic secretome. Mol Hum Reprod.

